# Commentary: Comparative efficacies of various corticosteroids for preventing postextubation stridor and reintubation: a systematic review and network meta-analysis

**DOI:** 10.3389/fmed.2023.1289321

**Published:** 2023-11-17

**Authors:** Yao Sun, Huiying Zhao, Ye Ma, Youzhong An

**Affiliations:** ^1^Department of Critical Care Medicine, Peking University People's Hospital, Beijing, China; ^2^Department of Anesthesiology, Peking University People's Hospital, Beijing, China

**Keywords:** corticosteroids, extubation, stridor, commentary, meta-analysis

## Introduction

The recent article by Feng et al. has drawn attention to the management of intubation and extubation in the field of critical care anesthesia (1). The article reviews some random control trials on the effect of different types of hormones on the prevention of post-intubation extubation stridor and reintubation. Using the PICOS principle, all relevant literature was reviewed and analyzed, then summarized for each trial. A net meta-analysis was performed to evaluate the quality of the articles and a subgroup analysis was attempted, resulting in 11 RCTs being included. The results of the study showed the superiority of using cortisol.

## Discussion

In our clinical work, especially in tonsil and adenoidectomies in children, we have observed that many patients present with post-extubation stridor. So this side effect caught our attention. Through a brief literature search, we learned of the earlier and more frequent use of hormones to prevent the onset of this side effect. This team's meta-analysis of the literature did a good job of summarizing the results of the research over time and effectively demonstrated that hormones are a more effective means of relief.

However, it is unfortunate that the type of disease the patient has is not well-defined in this article. Regarding hormonal prevention of post-extraction stridor, most of them are still focused on the patients in the intensive care unit, and only this study by Amoozadeh and Beigmohammadi suggests the effect of hormones before extubation in general anesthesia procedures (2). We made the following list of relevant postoperative extubation stridor after a literature search (Table 1).

**Table 1:** The review of prophylaxis of post-extubation stridor.

**Intervention**	**Dose**	**Route of administration**	***n***	**Primary author**	**Age**	**Type of surgery and technique**	**Anesthetic details**	**Time range**	**Type of research**	**Hospital and country**
Supine/ prone	/	/	242	Xiang (3)	NA	ERCP	NA	NA	A randomized controlled trial	NA
Supine/ lateral	/	/	92	Jung (4)	3–12 yr	NA	NA	NA	A randomized clinical trial	Yungpook National University; Korea
Albuterol spray	2 puffs	inhaled	120	Maddah (5)	52.34 ±8.95 yr	NA	NA	In 2021	A bouble-blind randomized clinical trial	5 Azar Educational Hospital in Gorgan; Northern Iran
Dexamethasone	8 mg TID first 24 h; 4 mg BID next 24 h	iv	110	Amoozadeh (2)	52.1 ± 14.1 yr	Neck surgery	NA	April 2021 to July 2021	An observational prospective cohort study	Imam Khomeini Hospital's; Iran
Remifentanil/ ambroxol hydrochloride/ budesonide suspension	Remifentanil/ 0.5 μg/g; ambroxol 15 mg hydrochloride; budesonide suspension 0.5 mg	iv/ inhaled	46	Yang (6)	NA	Removing the tonsil under general anesthesia and adenoidectomy under nasal endoscope	NA	NA	A randomized clinical trial	Cangzhou Central Hospital; China
Tracheal tubes	/	/	2246	Weiss (7)	1.93 (1.48) yr in the cuffed and 1.87 (1.45) yr in the uncuffed	ENT, Head surgery, Cleft, Thoracic, Abdominal, Laparoscopy, Urology, Limb, Cardiac catheterization, Gastroenter, Radiology, and Others	NA	NA	A prospective, randomized, controlled multi-center trial	University Children's Hospital Zurich; 24 European pediatric anesthesia centers; Switzerland
Bloodletting acupuncture	/	/	60	Saghaei (8)	33.8 (18.6); 38.37 (15.5) (month)	Scheduled for elective surgery	Atropine 10 μg.kg^−1^; Midazolam 25–100 μg.kg^−1^; thiopental 5 mg.kg^−1^; Fentanyl 1 μg.kg^−1^; Succinylcholine 2 mg.kg^−1^; halothane	NA	A double-blind, randomized trial	NA
Cuffed vs. uncuffed endotracheal intubation	/	/	40	Nishat (9)	3–10 yr	Oral surgeries,	Sevoflurane	February to December 2019	A randomized controlled study	NA
Lidocaine	topical 4 mg/kg of 2%; intravenous 1 mg/kg of 2% iv	/	134	Koç (10)	7.1 ± 1.7; 6.7 ± 1.3 yr; 7.6 ± 2.4 yr; 6.3 ± 0.8 yr	Tonsillectomy and/or adenoidectomy	Atropine, 0.015 mg/kg, meperidine, 1 mg/kg; gas mixture of N2O, O2, and halothane	NA	A randomized controlled trial	NA
Dexmedetomidine	Dexmedetomidine 2.0 μg/kg	im	100	Ambesh (11)	35.64 ± 14.93 yr; 38.08 ± 12.58 yr	Laminectomy for PIVD	Midazolam (0.05 mg/kg); Propofol (1.5–2.0 mg/kg); fentanyl (2.0 μg/kg); Vecuronium bromide (0.12 mg/kg), mixture of 50% air in oxygen and isoflurane (1%).	September 2014 to September 2016	A double blind placebo controlled study	Departm NA Anesthesiology, Sanjay Gandhi Post Graduate Institute of Medical Sciences; Lucknow
Sealing cuff pressure	/	/	60	Al-Metwalli (12)	8.35 ± 1.63 yr; 8.45 ± 1.76 yr; 8.2 ± 1.54 yr	Dental surgery	N2O free general anesthesia; 0.2 mg/kg oral diazepam; fentanyl 2 μg/kg and propofol 2.5 mg/kg.	NA	A prospective controlled, randomized, blinded study.	NA
Flexible laryngeal mask	/	/	90	Naguib (13)	3.24 ± 0.857 yr; 3.13 ± 0.815 yr	NA	Atropine 0.01 mg/kg; Fentanyl 1 μg/kg; Sevoflurane 3.5% in oxygen to air of 1:1	July 2018 to April 2019.	A randomized trial	Department of Pediatric Surgery, Tanta University Hospitals; Egypt

In addition, we also noted that some of the differences in the induction and maintenance of anesthesia may also affect the extubation of patients, such as dexmedetomidine (11). Feng's et al. also pointed out that some of the data in the literature is missing and incomplete, and we believe that this part of the data is also often a relatively critical part of the data that affects the results, for example, the use of some sedative and antagonistic drugs (1). It is hoped that there will be a careful description of randomized controlled trials in the future, which will also help us to have a more comprehensive and accurate understanding of drug efficacy.

At the same time, we found two studies on extubation position and bloodletting acupuncture interesting, both of which showed that extubation in the lateral position may reduce the probability of postoperative dyspnea (3, 4, 8).

## Author contributions

YS: Writing – original draft. HZ: Funding acquisition, Writing – review & editing. YM: Writing – original draft. YA: Funding acquisition, Supervision, Writing – review & editing.
